# The genomics revolution comes to the immunopeptidome

**DOI:** 10.1038/s41435-023-00244-5

**Published:** 2023-12-22

**Authors:** Peter M. Bruno

**Affiliations:** 1 Department of Urology, University of California, San Francisco, San Francisco, CA 94158 USA; 2 Helen Diller Family Comprehensive Cancer Center, University of California, San Francisco, San Francisco, CA 94158 USA

**Keywords:** MHC class I, Antigen presentation

Next-generation sequencing (NGS) has revolutionized nearly every area of biology, allowing for exponential increases in information across genomes, transcriptomes, translatomes, etc. Meanwhile, the development of high-throughput screening technologies, such as CRISPR-Cas9 knockout screens, have allowed biologists to better interpret the new “omics” data and understand which genetic changes are relevant to a given phenotype. Yet, the genomics revolution has yet to fully penetrate the field of immunology, where the staggering diversity of immune receptors and their potential targets, has made systematically assigning genetic sequence to disease pathology much more challenging.

For most biologists, antibodies and T-cell receptors (TCRs) are first to come to mind when thinking of the genetic diversity of the adaptive immune system, and rightfully so. The combinatorial complexity of recombination of various genomically encoded V-, D- and J-segments, compounded by junctional diversity, and somatic hypermutation in the case of antibodies, results in theoretical complexities exceeding 10^20^ possibilities. However, these sequences are more easily ascertained thanks to advances in single-cell sequencing. Without an NGS-based readout, arguably then the greater challenge is understanding the diversity of peptide:MHC (pMHC) complexes that are responsible for presenting antigens to TCRs.

The Major Histocompatibility Complex (MHC) gene locus is the most polymorphic region of the human genome, and these polymorphisms are associated with response to infections, predisposition to autoimmune disease, and cancer treatment outcomes [1]. That is because the gene products of this region, MHC-I and MHC-II, display different short peptide epitopes to communicate to the TCRs of CD8^+^ or CD4^+^ T-cells, respectively. Collectively, the peptides associated with MHC molecules are referred to as the immunopeptidome. Over a thousand unique alleles exist for each MHC-I gene, and each allele is capable of presenting ~10^9^ unique peptides to T-cells, yet, to date, fewer than 10^6^ peptides are confirmed MHC-I ligands [2]. Thus, a significant barrier to our understanding of the adaptive immune system is the staggering potential diversity of the immunopeptidome.

Mass spectrometry (MS) of peptides eluted from MHC immunoprecipitation has been the most dependable and accurate methodology for identifying MHC ligands, with large-scale experiments capable of identifying ~1,000 peptides per experiment. Thanks to these efforts, we now understand basic allele binding preferences for over 100 common alleles. However, a key limitation is that MS-based approaches must sample peptides from the entire cellular proteome, and thus cannot easily be used to specifically query pathogen- or neo-antigen-derived peptides. Furthermore, standard sample preparation techniques and peptide identification pipelines have limited the extent to which post-translation modifications (PTMs) can be reliably identified. However, recent targeted MS-based efforts, and bioinformatics advances, have made progress in neo-antigen [3] and PTM [4] MHC-I ligand identification, respectively.

Biochemical reconstitution of the pMHC complex has been the primary means of testing specific peptides for MHC binding. Unlike MS, biochemical reconstitution also has the capability of providing information on peptide affinity for MHC. The main drawback of biochemical reconstitution is that throughput is limited by the cost and effort of synthesis of the pMHC complex components. For instance, solid phase peptide synthesis used to generate the candidate MHC-I ligands takes 2-3 weeks and costs ~$100 per peptide. Despite this, the technique has been used successfully for decades to identify MHC-I binders.

**Fig. 1:**
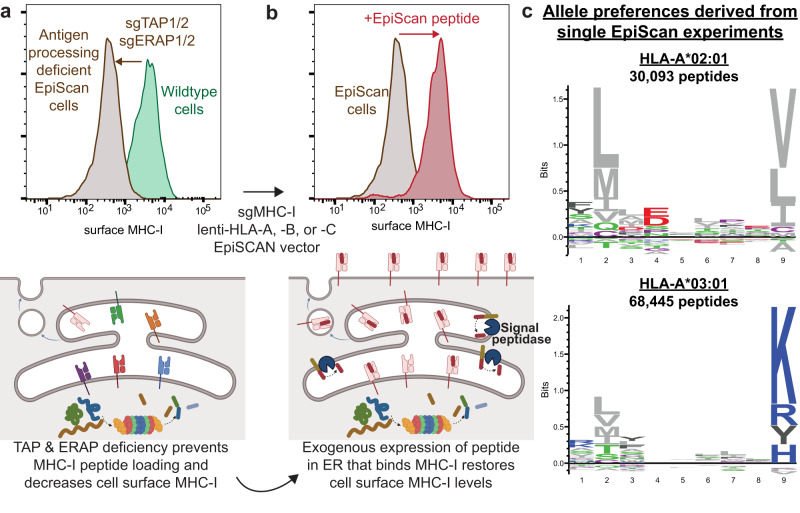
Diagram of the EpiScan approach and logoplots of allele binding preferences. EpiScan relies on the principle that MHC-I molecules are only trafficked to, and maintained on, the cell surface after stably binding a suitable peptide in the ER. **a** The ER is depleted of short peptides capable of binding MHC-I after knocking out ERAP1/2 and TAP1/2, thus MHC-I is not detectable on the cell surface. **b** Without endogenous short peptides in the ER, we use the lentiviral EpiScan vector to express an exogenous peptide of choice into the ER. If that peptide binds MHC-I we see increased cell surface MHC-I. The cells expressing MHC-I ligands can then be sorted via flow cytometry and the peptide sequence determined by NGS. **c** EpiScan-derived logoplots of two common MHC-I alleles, A*02:01 and A*03:01.

To address the lack of MHC-I ligand identification approaches that are both high-throughput, and capable of testing specific peptides, we developed EpiScan [5] (Fig. 1). To create EpiScan, we used CRISPR-Cas9 to create HEK293T cells deficient for endogenous MHC-I expression and devoid of short peptides in the endoplasmic reticulum (ER). Therefore, when a short peptide is supplied to the ER, and if it binds an exogenously expressed MHC-I allele, it will traffic to the cell surface, and we can detect the increased surface MHC-I by flow cytometry.

Importantly, with EpiScan, the candidate MHC-I ligands are encoded via DNA, and thus the identity of peptides that successfully bound MHC-I are determined via NGS. DNA encoding of the peptides also allows us to take advantage of inexpensive DNA oligonucleotide synthesis to design custom peptide libraries comprising hundreds of thousands of peptides derived from proteins of our choosing. Now, using EpiScan, we are able to apply the full advantages of the genomics revolution to the immunopeptidome. With EpiScan, we can perform screens for MHC-I ligands amongst predetermined starting pools comprising >100,000 peptides, a dramatic improvement in the scale and specificity of potential epitope determination.

We exploited this capability to screen the entire SARS-CoV-2 proteome across 11 different MHC-I alleles, identifying conserved, high-affinity, T-cell reactive epitopes. For three alleles used in further screening efforts, we identified more MHC-I ligands than identified by all previous efforts combined. Because the readout of EpiScan is genetic and unbiased by the biochemical properties of individual peptides, these screens uncovered a surprising role for cysteine that increases the number of potential epitopes by as many as 2.4 million per allele. Using these data, we created EpiScan Predictor (ESP), which, unlike preceding predictors, represents cysteine-containing peptides at percentages consistent with its frequency in the proteome.

Going forward, we aim to continue to leverage the programmable nature of EpiScan to identify MHC-I ligands that would be difficult to identify via traditional approaches. For instance, peptides longer than nine amino acids are relatively uncommon, and as a result, difficult to predict by peptide binding algorithms due to lack of training data. Designing peptide libraries exclusively composed of longer peptides would rapidly expand longer ligand training datasets. Furthermore, discovery of more disease-specific MHC-I ligands will aid in the design of personalized vaccines and broaden the potential target landscape for new immunotherapeutics. Retrospective studies of the potential immunopeptidome of cohorts of immune checkpoint blockade (ICB) recipients can elucidate determinants of ICB response. Autoimmune diseases, including adverse drug reactions, with clear genetic linkage to MHC-I can be readily studied via EpiScan to find MHC-I ligands that play a role in disease pathology.

In its current form, EpiScan cannot test PTMs. We plan to address this by using genetic code expansion to encode and test unnatural amino acids to better understand PTM binding to MHC-I. Additionally, EpiScan does not account for peptide processing, such as by the proteasome or other proteases. However, prior knowledge of peptide processing preferences by these enzymes can be used as a filter for selection of potential T-cell epitopes.

By designing a functional genetics approach to MHC-I ligand determination, we can take full advantage of the genomics revolution for exploring and exploiting the immunopeptidome. We believe the programmable nature of EpiScan, paired with its ease of use and increase in scale, will lead to substantial improvements in our understanding of the immunopeptidome and how best to optimize existing immunotherapies and design new ones.
